# Gunshot Wound of the Brain

**Published:** 1846-03

**Authors:** 


*Gunshot Wound of the Brain, attended with extensive Cranial Fracture, and followed by complete Recovery.*—The following very remarkable case is related by Mr. Ford, of the Madras Medical Establishment, in the L. & E. Monthly Journal of Medical Science, Sept., 1845.—On the morning of the 31st January, 1845, a lad about 15 years of age was brought into hospital with a gunshot wound of the head. He was quite insensible, and breathing stertorously upon admission. Examination, after shaving the head, disclosed the following amount of injury:

The ball had entered the head on a level with, and three-fourths of an inch anterior to, the summit of the right ear, and had made its escape through the left os frontis, an inch and a quarter above the centre of the eyebrow, fracturing the skull irregularly between both wounds, in such a manner as to implicate and depress the right orbit. The right os frontis, over its sinus, had likewise met with a comminuted fracture, which gave to the touch the crepitating sensation of a broken egg-shell. In addition to this varied fracture, one proceeded upwards and backwards, from the spot where the ball entered to the lambdoidal suture. The vortex was raised and inclined over to the left side, to such an extent as to enable the finger to be laid in the sulcus formed by the gaping margins of the fracture, especially between one wound and the other, and considerable pressure failed to approximate appreciably the edges. The right eye was completely closed by tumefaction, and the sinking of the orbit; strabismus had occurred in the other, and its pupil was but a speck. From the wound in the forehead, blood was trickling, and much had evidently escaped from it, as large clots were still adherent to his face. No spiculæ of bone were detected in either wound. The hemorrhage was soon checked by a cold application, which was continued to the head; the orbit was raised to its proper position; and saline and camphor mixture administered.

The following is a brief abstract of the most prominent symptoms, and treatment of this extraordinary case:

On the 3d February, he became in a slight degree sensible, and answered, Yes, (in Hindostanee,) to each and every question. The brain had gradually protruded since admission, through the outlet at the forehead, and on this date a mass larger than a walnut, and about half an ounce in weight, consisting of cortical and medullary substance, was excised. Pulse 130, and of moderate strength. The saline mixture and lotion were continued, and a purgative draught exhibited.

On the evening of the 5th, delirium, incoherent speech, and other symptoms of cerebral inflammation supervened. The head was freely leeched, and the sero-sanguinolent contents of a large swelling at the posterior extremity of the fracture, contiguous to the occiput, were evacuated by free incision. The whole scalp was puffy. Pulse 160, and distinctly computable. The purgative draught was repeated, and the mixture and lotion continued.

6th. The leeches were again applied.

7th. Some thin semi-transparent pus was seen oozing through the three wounds, including the scarification at the back of the head. Pulse 140, and soft. Medicines continued.

8th. Discharge more copious and purulent, and pieces of brain, unaccompanied by any fragments of bone, were passed from the wounds caused by the bullet. Delirium had increased. Was directed to take half a grain of the bi-sulphate of quinine, three times a-day; and mutton broth frequently. The wounds were kept open by dry lint.

9th. Stools and urine were passed involuntarily. The delirium continued unabated. The discharge from the wounds was free and healthy.

10th. A puncture was made in the right upper palpebra, near the inner canthus, and exit afforded to about two ounces of thick fetid pus. He remained insensible, and the excretions were voided without his will.

12th. Tne pus, somewhat fetid; has been plentifully poured forth from the four openings, which have been kept open by pledgets of lint. The puncture made the day before yesterday in the palpebra, is found to communicate now with the wound in the forehead. Pulse feeble, and 130. To have his broth as before. and a grain of the bi-sulphate of quinine thrice a-day.

14th. He appeared to know what was said to him, and the left eye, which he now opened and directed to any object, seemed to possess perfect vision. Made signs for the bed-pan. Discharge free. Delirium at night. The treatment continued.

16th. He evidenced by his acts more perception and intelligence. Discharge healthy and profuse. He took two grains of bi-sulphate of quinine every six hours. Allowed as much animal food as he wished.

17th. Much confusion, anxiety, and irritability of manner existed to-day. Frequent paroxysms of incoherence during the night. Pulse 130, and small. A quarter of a grain of acetate of morphia was ordered to be taken with each dose of the quinine.

19th. Was calmer. Discharge diminished. No incoherency. Treatment continued.

21st. Discharge gradually decreased. Labored under symptoms of fatuity, and replied only "yes," to any question. Had compresses applied to the right os frontis and to the occiput, (the incision near which had almost healed,) and adhesive plaster to the ball wounds. Continue the quinine and morphia. The bowels remained free, without the use of any purgative.

28th. Since last date, the wound at the occiput has healed; and the discharge is now slight from the other three.—Constitutional irritability has been much allayed. Slept and ate well. Was quite idiotic, and constantly attempted to remove his bandages. Opened his right eye, of which the vision has been injured. The motor power only of the left side has been partially lost. The mouth is drawn to the right. He cannot project his tongue; and his endeavors to do so caused laughter. The doses of morphia and quinine were augmented respectively to half a grain, and three grains. Nitrate of potash, and nitric acid, were likewise prescribed. Compresses, &c., were applied as before.

March 5th. Some dawning of intellect was perceptible to day, and a word of rebuke had a temporary effect in checking his propensity to remove his bandages and destroy his bed-clothes. Vision was perfect, and the axes of both eyes were simultaneously directed to any object. Has been disposed to speak. Urine passed in abundance. The exposed surface of the brain at the frontal wound has numerous small vessels traversing it, and it has put on the character of mucous membrane, yet without any attachment to the surrounding integuments of the forehead. Pulse large, and 100.

10th. Has decidedly improved in his mental capabilities, and has recognized his friends, including an officer who came to see him, and whose name he distinctly pronounced. When any question has been put to him with a caution, he has replied sensibly. The pieces of the right os frontis have consolidated with one exception, and an angle of a small fragment projects under the integuments at the upper part of the forehead. Contrary to my expectation, no pus has ever been discharged by the nose. The space between the edges of the fracture, posterior to where the ball entered, has closed, but in no way between that and the wound of the forehead. The discharge has been trivial. Two small squamæ of bone have been removed from the wound near the ear. Has walked a little with assistance. No improvement in the use of his left upper and lower extremities; they are employed at will, but feebly; their sensibility, however, has never been involved. The new membrane, remarked as covering the surface of exposed brain, has thrown out exuberant granulations, which have been with difficulty kept down to the level of the frontal teguments, by the liberal application of lunar caustic. Ordered to take, three times a-day, an ounce of compound infusion of gentian, half a grain of morphia, and four drops of muriatic acid. Compresses continued.

To render this report as concise as possible, I conceive I may, without any disadvantage to it, conclude by recording, that from this date, a progressive amendment to complete and perfect restoration of the patient's health of mind and body took place, under a continuance of the treatment above noted. On the 3d of April he was discharged from the hospital. The vertex is permanently settled to the left side. The skull has not united for about two inches over the temple, where, and at the cicatrix of the frontal wound, the pulsations of the brain can be conspicuously observed. A ridge exists where union has occurred in other parts of the fracture. The wound in front of the ear has closed with ossific matter. The right eye cannot be opened quite so widely as the left, and there exists some thickening of the orbital margin of the frontal bone. The right commissure of the mouth is but slightly retracted. His sleep is refreshing, his appetite hearty, his secretions normal, and he is fleshy and strong. His memory is tenacious, and he can summon to it any bygone event to which he has been a party, with accuracy and promptitude; and he is subject to no hallucination. In short, his senses of hearing, smell, taste, sight, and touch, have not been one tittle deteriorated by the violent and extensive nature of the injury to the brain.

He presents himself occasionally for examination; and he has just now paid me a visit. I cannot learn that his disposition has been in the least altered by his accident and consequent loss of cerebral substance. He is good-tempered and cheerful, which his friends say he has been from his infancy.—*Amer. Jour. of Med. Sciences.*

